# The impact of thyroid disorders on the clinical outcome of assisted reproductive techniques: a systematic approach over the last 10 years

**DOI:** 10.5935/1518-0557.20230024

**Published:** 2023

**Authors:** Clara Andrade Teixeira, Ana Normélia Pereira de Morais, Gabriel Acácio de Moura, Isadora de Almeida Gomes, Sabrina Vieira de Souza, Mônica Odilha Magalhães Dias, Ana Beatriz Rocha Lima, Paula Bruno Monteiro

**Affiliations:** 1 Christus University Center (UNICHRISTUS), Fortaleza-CE, Brazil; 2 Laboratory of Manipulation of Oocytes and Preantral Follicles, Post-Graduate Program in Physiological Sciences (PPGCF), State University of Ceará (UECE), Fortaleza-CE, Brazil; 3 Oswaldo Cruz Foundation (FIOCRUZ), Eusébio-CE, Brazil

**Keywords:** autoimmune disease, hypothyroidism, cancer, fertility

## Abstract

**Objective:**

To verify from a systematic literature review the possible effects of thyroid diseases on assisted reproduction techniques.

**Data sources:**

The studies were analyzed from the PubMed, Cochrane Library, LILACS databases.

**Selection of studies:**

The articles selected for the review included: cross-sectional studies, cohort studies, and clinical trials that addressed the proposed theme and which were published within the period stipulated from January 1, 2012, to March 5, 2022, in English, Portuguese and Spanish. These would later have to go through stages of inclusion as a framework of the type of study and exclusion criteria that were review articles, case reports, abstracts, articles with animal models, and duplicate articles and letters to the editor.

**Data collection:**

Author’s name; Number of patients; Clinical outcome; Use of drugs; Control group (in case it had); Clinical outcome.

**Data synthesis:**

In *in vitro* fertilization and intracytoplasmic sperm injection it was verified that thyroid diseases can lead to effects such as a reduction in the rate of recovered oocytes, a decrease in the number of embryos, lower pregnancy rates, and increased chances of congenital anomalies in these patients and a reduction in the rate of implantation. Levothyroxine can increase the number of cycle cancellations.

**Conclusions:**

Thyroid diseases may have deleterious effects on the clinical outcome of *in vitro* fertilization and intracytoplasmic sperm injection.

## INTRODUCTION

Infertility has always been considered a primary challenge for reproductive medicine, which is defined as a flaw in conception after regular unprotected sexual relations for 2 years in the absence of known pathologies ( Szamatowicz & Szamatowicz, 2020 ). Estimates from the World Health Organization (WHO) suggest that infertility is present among about 48 million couples and 186 million individuals worldwide ( WHO, 2020 ). Another worrisome factor is that infertility is a relatively common problem that affects about 15-20% of couples of reproductive age ( Sihag *et al* ., 2018 ).

Currently, even though there is a substantial increase in Assisted Reproduction Techniques (ART) such as *In Vitro* Fertilization (IVF), Intracytoplasmic Sperm Injection (ICSI), and Intrauterine Insemination (IUI), there are large amounts of both physiological and environmental influences that can lead to failures in these procedures ( Ricci *et al* ., 2020 ). A fine example of this, Di Mario *et al* . (2019 ) observed in a study with 86 women of reproductive age with Systemic Lupus Erythematosus a reduction in ovarian reserve associated with the severity of the disease and sequential therapy with cyclophosphamide and conventional disease-modifying anti-rheumatic drugs such as methotrexate, azathioprine, mycophenolate mofetil and cyclosporine. It is noteworthy that there is an increase in studies aiming at verifying whether metabolic disorders are negatively associated with clinical outcomes of ARTs amid these disorders are Thyroid Diseases (TD) ( Heber & Ptak, 2021 ).

TDs comprise a set of functional, inflammatory, autoimmune, and neoplastic benign pathologies, characterized by a wide range of physical and mental symptoms ( Leso *et al* ., 2020 ). In most cases, these symptoms are associated with two main thyroid hormones: thyroxine (T4) and triiodothyronine (T3), that affect various physiological processes such as maintaining body temperature, digestion, and vital functions such as heart and respiratory rate ( Green *et al* ., 2021 ). These thyroid hormones have as their main regulator the glycolipid called pituitary hormone thyrotropin (TSH), produced by the anterior pituitary gland, which will also present changes in its serum level in cases of pathology ( Mullur *et al* ., 2014 ). Literature also infers a direct activity of thyroid dysfunction in the reproductive system, since sex gametes (especially oocytes) have thyroid hormone receptors on their cell surface. In addition, there is an indirect activity of thyroid hormones in the interruption of the Gonadotrophin release Hormone (GnRH) essential to produce these gametes ( Öztürk Ünsal *et al* ., 2021 ).

Given the above, there is a need to verify the possible effects of TD on the clinical outcomes of ARTs to establishing protocols that will improve the clinical management of patients with TDs to increase the success rates of these techniques ( Unuane & Velkeniers, 2020 ). In addition, there is a shortage of studies that recommend or contraindicate the use of routine therapies specific to women with TD who have impaired fertility and are seeking to become pregnant with the aid of ARTs ( Wadhwa *et al* ., 2020 ). Therefore, the present study aims to evaluate from a systematic literature review the deleterious activity of TDs in the clinical outcome of ARTs, in addition to investigating the influence of the most used drugs for treatment in the protocols of ART.

## MATERIALS AND METHODS

### Type of study

The present study is a systematic literature review. The Preferred Reporting Items for Systematic Reviews and Meta-Analyses (PRISMA) rules guide was used for its Fulfillment. The entire protocol was submitted and registered at the National Institute for Health Research (PROSPERO) under protocol number (CRD42022320836).

### Search strategy

In order to search the articles in the present study, descriptors were initially checked by medical subject headings (MESH). After selection, the descriptors were allocated in combination with Booleans in three different studies: (1) autoimmune thyroiditis OR thyroid diseases AND *In vitro* fertilization, (2) autoimmune thyroiditis OR thyroid diseases AND intracytoplasmic sperm injection, (3) autoimmune thyroiditis OR thyroid diseases AND artificial insemination. This search strategy was performed in three distinct databases: PubMed, Cochrane Library, and LILACS.

### Eligibility criteria for studies

For the selection of studies, the following eligibility criteria were adopted:

1. **Studies:** Clinical trials, cohort studies and cross-sectional studies that addressed the theme proposed by the authors;
2. **Publication period:** The studies evaluated in this review were published between January 1, 2012 and March 5, 2022;
3. **Language of the studies:** Articles were selected in the following languages: English, Portuguese and Spanish;
4. **Intervention performed:** Studies that addressed the rate of oocyte uptake and maturation, fertilization, clinical pregnancy, live birth rate and hormonal level;
5. **Outcome:** Obtaining the effects of TD on the clinical outcome of ARTs.

### Criteria for selection of studies

- *Inclusion criteria*
  - Cohort studies;
  - Cross-sectional studies;
  - Clinical trials.
- *b Exclusion criteria*
  - Out-of-scope studies;
  - Review articles;
  - Letters to the editor;
  - Abstracts;
  - Animal studies;
  - Case reports;
  - Duplicate articles.

### Assessment of methodological quality

To reduce the chance of bias among the articles, the methodological quality assessment of the studies was performed from the Newcastle Ottawa analysis tool that allows evaluating the methodological quality of observational studies ( table 1 ).

**Table 1:** Quality assessment of the studies by the Newcastle-Ottawa scale

Study	NOS Items Score								
	**Criteria**								
**Criteria**	**Selection**				**Comparability** **1a**	**Results**			**Total**
	**1**	**2**	**3**	**4**	**1**	**2**	**3**	
Zhong *et al* ., 2012	1	1	1	1	1	1	1	1	8
Scoccia *et al* ., 2012	1	1	1	1	1	1	1	1	8
Busnelli *et al* ., 2013	1	1	1	1	1	1	1	1	8
Tan *et al* ., 2014	1	1	1	1	1	1	1	1	8
Chai *et al* ., 2014	1	1	1	1	1	1	1	1	8
Unuane *et al* ., 2016	1	1	1	1	1	1	1	1	8
Neto *et al* ., 2016	1	0	1	1	1	1	1	1	7
Unuane *et al* ., 2017	1	1	1	1	1	1	1	1	8
Cai *et al* ., 2017	1	1	1	1	1	1	1	1	8
Nacak *et al* ., 2018	1	1	1	1	0	1	1	1	7
Pekman *et al* ., 2019	1	1	1	1	1	1	1	1	8
Ke *et al* ., 2020	1	1	1	1	1	1	1	1	8
Poppe *et al* ., 2020	1	1	1	1	1	1	1	1	8
He *et al* ., 2020	1	1	1	1	1	1	1	1	8
Gungor & Gungor, 2021	0	1	1	1	1	1	1	1	7
Zhuang *et al* ., 2021	0	1	1	1	1	1	1	1	7
Rao *et al* ., 2021	1	1	1	1	1	1	1	1	8
Huang *et al* ., 2021a	1	1	1	1	1	1	1	1	8
Huang *et al* ., 2021b	0	1	1	1	1	1	1	1	7
Huang *et al* ., 2021c	0	1	1	1	1	1	1	1	7

Subtitle: Selection 1: Representative of the exposed cohort; Selection 2: Selection of the non-exposed cohort; Selection 3: Ascertainment of exposure; Selection 4: Outcome of interest not present at the start of the study; Comparability 1a and 1b: Comparability of cohorts based on the design or analysis; Results 1: Assessment of outcomes; Results 2: Follow-up of cohorts; Results 3: Adequacy of follow-up of cohorts.

### Data collection

After searching, the articles were assessed through title and abstract. Excluding those that did not fit as mentioned above. When the articles met the criteria established in the review, they were evaluated by seven authors (TCA, MGA, MANP, GIA, SVS, DMOM and LABRL). If they agreed on the clinical outcome, data would be tabled. If a divergence occurred, data would be re-analyzed by a fourth author specialist in the area (MPB).

### Data extraction

Soon after collection, these articles were read to obtain the following data:

- Name of the author;
- Number of patients;
- Clinical outcome;
- BMI;
- Use of drugs;
- Control group (in case it had)
- Clinical outcome.

## RESULTS

### Study characteristics

**Figure 1:**
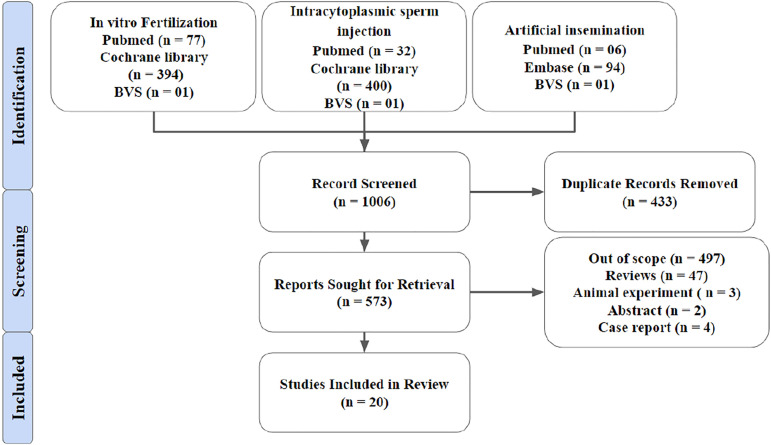
Methodological screening.

In total, 1006 articles were found using the aforementioned methodology, selecting 20 articles among them for the preparation of this review, as shown in Figure 1 . The number of patients inserted in the articles used in the present review totaled 5,653, where the mean age varies from 20 to 38 years and with body mass index (BMI) from 20.5 to 25 kg/m^2^. Data is presented in Table 2 .

**Table 2:** Thyroid diseases and clinical outcomes in assisted reproduction

Thyroid disease	Author	Number of patients	Age/ TSH levels	BMI (kg/m^2^)/ TSH levels	Drug	Control group	Clinical outcome
Thyroid autoimmunity	Zhong *et al* .,2012	90	32.8y	20.5	-	Euthyroid (negative self-immunity)	( *IVF* + ICSI) ↓ Fertilization; ↓ Viable embryos; ↓ Pregnancy rate
	Tan *et al* .,2014	835	31.4y	23.7	-	Euthyroid (negative self-immunity)	(ICSI) No statistical differences
	Chai *et al* .,2014	122	35.2y	21.1	-	Euthyroid (negative self-immunity)	( *IVF* + ICSI) No statistical differences
	Unuane *et al* .,2016	333	33.4y	25.2	Levothyroxine	Euthyroid (negative self-immunity)	( *IVF* + ICSI) No statistical differences
	Unuane *et al* .,2017	187	33.83y	23.8	-	Euthyroid (negative self-immunity)	(IIU) No statistical differences
	Nacak *et al* .,2018	Group IOP: n=150 ovarian aging group in old age:n=150	group IOP: 30.5y ovarian aging group in advanced age:36.8y	-	-	-	(ICSI) group IOP:↓ Pregnancy rate
	Ke *et al* .,2020	1075	31	23.4	-	Euthyroid (negative self-immunity)	( *IVF* + ICSI) ↑ Congenital anomalies
	Poppe *et al* .,2020	25	36.5	24.6	-	Euthyroid (negative self-immunity)	( *IVF* + ICSI) ↓ Number of revored oocytes
	He *et al* ., 2020	0.3 <TSH<2.5 mIU/L:54 2.5<TSH<4.2 mIU/L:54	0.3<TSH<2.5mIU/L:30.8y 2.5<TSH<4.2mIU/L:30.3y	0.3<TSH<2.5mIU/L:23.1 2.5<TSH<4.2mIU/L:23.4	-	Euthyroid (negative self-immunity)	(ICSI) ↓ Embryo rate
	Gungor & Gungor,2021	39	31.6	25	-	Euthyroid (negative self-immunity)	( *IVF)* No statistical differences
	Zhuang *et al* .,2021	42	32.37	24.91	-	Euthyroid (negative self-immunity)	( *IVF* + ICSI) ↑ Premature delivery
	Huang *et al* .,2021a	778	20-38	22.6	-	Euthyroid (negative self-immunity)	( *IVF* + ICSI) ↓ Number of recovered oocytes
Hypothyroidism	Scoccia *et al* .,2012	21	33.3	-	Levothyroxine	Euthyroid	(FIV) ↓ Live births; ↓ Implementation; ↓ Clinical pregnancy
	Busnelli *et al* .,2013	137	35	22.9	Levothyroxine^*^	Euthyroid (Without Levothyroxine)	( *IVF* + ICSI) ↑ Cancellation of cycles
	Neto *et al* .,2016	TSH<2.5mUI/L:455 TSH:2.5-4.0mUI/L:162	TSH<2.5mUI/L:35y TSH:2.5-4.0mUI/L:35y	TSH<2.5mUI/L:24.2 TSH:2.5-4.0mUI/L 24	-	Subclinical hypothyroidism	( *IVF* + ICSI) No statistical differences
	Cai *et al* .,2017	200	28.8	22.7	LT-4	Euthyroid	( *IVF* + ICSI) No statistical differences
	Pekman *et al* ., 2019	387	27	24.4	-	Healthy control	(IIU) No statistical differences
	Rao *et al* .,2021	229	36	24.5	-	Euthyroid	( *IVF* + ICSI) No statistical differences
Thyroid cancer	Huang *et al* .,2021b	Total thyroidectomy:26. Partial thyroidectomy:38	Total thyroidectomy:34.9y Partial thyroidectomy:33y	Total thyroidectomy:23.3. Partial thyroidectomy:23	-	Healthy control	( *IVF* + ICSI) ↓ Live births
	Huang *et al* .,2021c	64	33.8	23.1	-	Healthy control	( *IVF* + ICSI) ↓ Number of recovered oocytes ↓ Fertilization ↓ Viable embryos

### Thyroid autoimmunity and clinical outcome of ARTs

Most studies analyzed the harmful effects of antithyroid antibodies on the clinical outcome of ARTs when compared to euthyroid patients with the absence of antibodies. In methodologies such as IVF and ICSI, it was verified a reduction in the rate of oocytes recovered in patients, a decrease in the number of viable embryos produced *in vitro* , lower pregnancy rates, and an increased chance of congenital anomalies in these patients ( Zhong *et al* ., 2012 ; Beydilli Nacak *et al* ., 2018 ; Ke *et al* ., 2020 ; Poppe *et al* ., 2020 ; He *et al* ., 2020 ; Zhuang *et al* ., 2021 ; Huang *et al* ., 2021a ). In studies without the use of IUI, significant differences were found in the clinical outcome of patients with anti-thyroid antibody positivity. In addition, in five studies, significant activity of thyroid autoimmunity in ARTs was not verified. ( Tan *et al* ., 2014 ; Chai *et al* ., 2014 ; Unuane *et al* ., 2016 ; 2017; Gungor & Gungor, 2021 ).

### Hypothyroidism and its impacts on ARTs

Our review found that this pathology can trigger problems in the clinical outcome of these patients. From the selected studies, as the main effects of this pathology, there was a reduction in the rate of implantation, clinical pregnancy and live births in patients undergoing *in vitro* fertilization and an increase in the cancellation of cycles in patients with combinatorial treatment of IVF and ICSI ( Scoccia *et al* ., 2012 ; Busnelli *et al* ., 2013 ). However, In the other studies, the presence of hypothyroidism was not associated with the clinical outcome of ARTs ( Coelho Neto *et al* ., 2016 ; Cai *et al* ., 2017 ; Pekcan *et al* ., 2019 ; Rao *et al* ., 2021 ).

### Thyroid cancer in ARTs

In this review, we verified only two studies that addressed the use of post-therapy ARTs for patients with thyroid cancer. In both studies, IVF and ICSI methodologies were cited, with a lower rate of oocyte uptake, fertilization, viable embryos, and live births rates in patients reported. ( Huang *et al* ., 2021b ; 2021c).

### Pharmacological use and its association with clinical outcome

We identified in our review that only four studies reported the use of drugs for treatment in thyroid comorbidity. One of the main drugs used was levothyroxine in three studies ( Scoccia *et al* ., 2012 ; Busnelli *et al* ., 2013 ; Unuane *et al* ., 2016 ). Lt-4 was used in a single study to treat hypothyroidism ( Cai *et al* ., 2017 ). In only one of these, an association was reported between the use of levothyroxine and a higher rate of cancellation of cycles ( Busnelli *et al* ., 2013 ).

## DISCUSSION

Thyroid dysfunctions comprise a set of diseases that can act directly on hormonal secretion and consequently on the physiological metabolism of the organism ( Piantanida *et al* ., 2020 ). The current literature infers that there are distinct thyroid hormone effects on ovarian function that may be correlated with the development of oocytes and embryos, however, there is still a great gap when these hormones are decompensated in thyroid dysfunctions ( Myneni *et al* ., 2021 ). Therefore, it is necessary to identify the effects of these diseases on reproduction, both for the provision of adequate immediate treatment and the improvement of the clinical results of patients with ongoing treatment ( Song *et al* ., 2019 ).

Three studies found in our review addressed the clinical outcome of thyroid autoimmunity and thyroid cancer in ARTs and a reduction in the number of oocytes captured was observed ( Poppe et al., 2020 ; Huang et al., 2021a ; 2021c ). This fact is supported by Huang *et al* . (2021d ), who verified the follicular fluid of patients positive for thyroglobulin antibody (TgAB) and thyroid peroxidase antibody (TPO) that had a higher rate of inflammatory cytokines that inhibit vessel angiogenesis, leading to the arrest of the tecal development and follicular atresia. In addition, recent evidence from the literature infers that thyroid stimulating hormone receptors (TSHr) are present in the granulosa and ovarian cells, and endometrium and, may act directly in the development and stages of oocyte maturation during folliculogenesis ( Mintziori *et al* ., 2016 ).

Our review also verified in three articles that thyroid diseases can lead to a reduction in the rate of fertilization and viable embryos in IVF and ICSI ( Zhong *et al* ., 2012 ; He *et al* ., 2020 ; Huang *et al* ., 2021c ). These findings were experimentally obtained by Lee *et al* . (2009 ) who immunized mice to stimulate TPO production and verify their effect on gestational and embryonic development and verified that mice that had TPO in serum had a lower rate of preimplantation embryos in a stage of 3/4 cells, in addition, an increase in embryonic absorption of antibodies. Another experimental study conducted by Tonyushikina *et al* . (2014 ) found that increased exposure to thyroid hormones can impair embryonic development in zebrafish, however, with their adjustment these embryos could continue in their development, leading to the hypothesis that the regulation of thyroid hormones is of utmost importance both for the development of oocytes embryos in patients submitted to ARTs with TDs.

Another result that drew a lot of attention was the reduction in implantation rates, pregnancy, and increase in the rates of congenital anomalies in patients with TDs ( Zhong *et al* ., 2012 ; Scoccia *et al* ., 2012 ; Beydilli Nacak *et al* ., 2018 ; Ke *et al* ., 2020 ; Huang *et al* ., 2021c ). These findings are also in agreement with the study conducted by Matalon *et al* . (2003 ), which verified the stimulation of TGA and TPO antibodies in BALB/c mice that in the stimulated group there was a significant increase in placental reabsorption. In addition, a high concentration of autoantibodies was found in the diluted placental supernatant indicating an antibody absorption rate that could explain gestational loss. Furthermore, as mentioned earlier in the study by Lee *et al* . (2009 ) a direct action of antithyroid antibodies was verified in the embryonic development of hyperstimulated mice, which would possibly justify the increase of congenital anomalies.

We also reviewed the activity of drugs used in TD therapies in the clinical outcome of ARTs. With this, a single study found that the use of levothyroxine is directly associated with cancellations in ART cycles ( Busnelli *et al* ., 2013 ). This finding is still widely discussed and divergent in the literature as in the study by Revelli *et al* . (2009 ), which found that patients with euthyroidism treated with levothyroxine exhibited a better response to *in vitro* fertilization. Another study previously conducted by Negro *et al* . (2006 ) confirmed these findings, verifying a possible improvement in the clinical outcome of patients using levothyroxine requiring further studies to prove this correlation.

As the main limitations of this review, we need to show that in most studies there was no comparison between patients with thyroid disorders concerning healthy control groups, which did not allow us to draw an accurate correlation as to the real impact on the clinical outcome of ARTs. In addition, the absence of studies that specifically focus on or do not consider the Nine articles showed no direct activity against thyroid diseases and the control groups ( Tan *et al* ., 2014 ; Chai *et al* ., 2014 ; Unuane *et al* ., 2016 ; 2017; Gungor & Gungor, 2021 ; Coelho Neto *et al* ., 2016 ; Cai *et al* ., 2017 ; Pekcan *et al* ., 2019 ; Rao *et al* ., 2021 ). These findings are in line with the study carried out by Reh *et al* . (2011 ) which verified both the prevalence of autoimmune thyroid diseases in patients with advanced age and their clinical outcome in ARTs, which did not significantly impact the outcome. a clinical trial of ARTs in patients, however, age was a limiting factor. Given this, the maintenance of a euthyroid state in the setting of desired fertility is already well recognized in terms of conception, pregnancy, and fetal development, although it is still unclear in the literature to what extent these thyroid comorbidities may cause adverse effects on fertility and TRAs ( Stuckey *et al* ., 2010 ).

Our data also showed that the authors were concerned about filtering for BMI in patients with thyroid dysfunction. It is already well described in the literature that TSH levels may be correlated with the BMI of these patients, as reviewed by Babić Leko *et al* . (2021 ). TSH and thyroid hormones. In addition, previous studies have already shown that BMI is also associated with clinical outcomes in a successful pregnancy, such as the one by Mitha *et al* . (2020 ) who found from a cohort containing live births that maternal BMI may be associated with seizures neonatal and gestational asphyxia. In another study, carried out by Qu *et al* . (2021 ), it was seen that gestational obesity is associated with higher risks of early and spontaneous abortion in the population undergoing ARTs. Use of drugs in the activity of possible adverse or beneficial effects for ARTs may have been a determining factor for the outcome of some studies. Given the above, TDs may have negative effects on the clinical outcomes of ARTs. Regarding IVF and ICSI, it was verified that TDs can lead to effects such as a reduction in the rate of oocytes recovered in patients, a decrease in the number of viable embryos produced *in vitro* , lower pregnancy rates, and an increased chance of congenital anomalies and, reduction in the rate of implantation. In the IUI, no activity was verified in the clinical outcomes of ARTs. The correlation between the use of drugs for thyroid diseases remains unknown in the literature, so further studies are needed to verify their potential impact.

### List of abbreviations:

- thyroid antibody peroxidase (TPOb)
- Thyroglobulin antibody (TGAb)
- Thyroid Diseases (TD)
- *In vitro fertilization* (IVF)
- Gonadotrofine Releasing Hormone (GnRH)
- Incytoplasmic sperm injection ( ICSI)
- Intrauterine Insemination (IIU)
- World Health Organization (WHO)
- Preferred Reporting Items for Systematic Reviews and Meta-Analyses (PRISMA)
- thyroid stimulating hormone receptors (rTSH)
- Assisted Reproduction Techniques (ART)
- Thyroxine (T4)
- Triiodine (T3)
